# Mortality before and during the COVID-19 pandemic in Manhiça district, Southern Mozambique

**DOI:** 10.1186/s12963-025-00449-y

**Published:** 2026-03-18

**Authors:** Charfudin Sacoor, Arsénio Nhacolo, Jonathan A. Muir, Edgar Jamisse, Beth Tippet Barr, Ariel Nhacolo, Chodziwadziwa Kabudula, Jean Juste Harrisson Bashingwa, Orvalho Augusto, Alberto Chaúque, Teodimiro Matsena, Arlindo Malheia, Aura Hunguana, Francisco Saúte, Solveig A. Argeseanu, Stephen Tollman, Esperança Sevene, Quique Bassat, Inácio Mandomando

**Affiliations:** 1 Centro de Investigação em Saúde de Manhiça (CISM), Manhiça, Mozambique; 2 Facultat de Medicina i Ciències de la Salut, Universitat de Barcelona (UB), Barcelona, Spain; 3 Emory University, Atlanta, GA USA; 4 Nyanja Health Research Institute, Salima, Malawi; 5 Wits University, Johannesburg, South Africa; 6 Faculdade de Medicina, Universidade Eduardo Mondlane, Maputo, Mozambique; 7 Department of Global Health, University of Washington, Seattle, WA USA; 8 South African Medical Research Council, Cape Town, South Africa; 9 Barcelona Institute for Global Health, Hospital Clínic de Barcelona, Barcelona, Spain; 10 Institució Catalana de Recerca i Estudis Avançats (ICREA), Barcelona, Spain; 11 Pediatrics Department, Hospital Sant Joan de Déu, Universitat de Barcelona, Esplugues, Barcelona, Spain; 12 CIBER de Epidemiología y Salud Pública, Instituto de Salud Carlos III, Madrid, Spain; 13 Instituto Nacional de Saúde, Maputo, Mozambique

**Keywords:** Excess mortality, COVID-19, HDSS, Manhiça, Mozambique

## Abstract

****Introduction**:**

Mozambique reported its first COVID-19 case in March 2020, and the pandemic exposed significant vulnerabilities in its healthcare system. Measuring mortality attributable to COVID-19 in Mozambique, has been challenging due to limitations in health information systems and incomplete death documentation outside health facilities. By mid-2023, a total of 2,234 deaths from 233,334 cases were confirmed but the figures can be much higher.

****Methods**:**

We conducted a trend analysis of mortality using data from the Manhiça Health and Demographic Surveillance System from the periods before (2016-2019) and during the pandemic (2020-2021) to measure sex differences in mortality patterns (life expectancy and mortality rates). Excess mortality ratios during the pandemic were assessed using time series analysis with COVID-19 a generalized additive model.

****Results**:**

From 2019 to 2020, the life expectancy in males increased 5.1%, from 61.3 (95% CI: 60.3–62.2) years to 64.4 (95% CI: 63.5–65.3) years, and 6.1%, from 69.3 (95% CI: 68.5–70.2) years to 73.5 (95% CI: 72.6–74.3) years in females. However, from 2020 to 2021, a decline was observed in both males and females. In males, it dropped 3.1% while in females the life expectancy dropped 3.5%. All-age male mortality rates decreased from 15.3 to 11.2 (26.8%) deaths per 1000 person-years from 2016 to 2020, then rose to approximately 13.4 in 2021. All-age female mortality experienced a similar trend, with an increase of 9.0% from 6.7 deaths per 1000 person-years in 2020 to 7.3 in 2021. During pandemic, the male elderly population (65+ years old) experienced the highest excess mortality in July 2021, reaching a ratio of 1.57 (CI: 1.37–1.84), whereas for females, the highest excess mortality among females was observed in the age group of 05–14 years, with a ratio of 1.86 (CI: 1.44 - 2.17) in January 2021 between the observed and expected deaths.

****Conclusion**:**

Mortality in Manhiça district declined from 2016 until 2019 but increased during COVID-19 pandemic with excess deaths in 2021, particularly among those aged 65 and older. This study highlights the value of robust health and demographic information systems in resource-limited settings for assessing public health impacts.

**Supplementary Information:**

The online version contains supplementary material available at 10.1186/s12963-025-00449-y.

## Introduction

COVID-19 originated in Wuhan, China, in December 2019 and escalated rapidly into a global pandemic that was officially declared by the World Health Organization (WHO) on March 11, 2020 [1]. This disease is caused by the Severe Acute Respiratory Syndrome Coronavirus 2 (SARS-CoV-2) [2, 3]. As of September 2023, the WHO estimates that there are more than 770 million COVID-19 cases globally, with more than 6.9 million reported fatalities [4]. Despite these staggering global numbers, significant regional disparities in data accuracy and reporting exist, particularly in Sub-Saharan Africa. Obtaining accurate COVID-19 mortality data in Sub-Saharan Africa has been challenging because of limited research, inadequate surveillance, limited healthcare infrastructure and resource constraints that compromise timely data collection and reporting [3, 5]. Data quality varies within and across countries due to factors such as testing capacity, underreporting of cases and deaths, and difficulties in accurately attributing deaths to COVID-19 [6]. Variations in definitions of COVID-19-related deaths among countries also affect data consistency [7]. Despite some countries establishing data platforms for reporting, resource limitations have led to delays and data gaps [6, 8]. These challenges were further compounded by the COVID-19 pandemic, which not only hindered data reporting but also interrupted mortality improvements in Sub-Saharan Africa by disrupting previous progress in healthcare infrastructure, reducing child mortality, and increasing life expectancy [9]. Mozambique reported its first COVID-19 case on 22 March 2020 and experienced four distinct waves of the pandemic by December 2021. The first wave emerged in early 2020, followed by a second wave driven by the Beta variant in early 2021. The third wave, dominated by the Delta variant, was the most severe, ending in early October 2021. By December 2021, the fourth wave, led by the Omicron variant, was most prevalent [10]. The COVID-19 pandemic caused significant disruptions to health service delivery, particularly in resource-limited. These disruptions stemmed from both the direct effects of the virus and the indirect pressures placed on already strained health systems, exposing critical gaps [11]. Mozambique’s National Health Service (NHS) exemplifies the constraints faced by low-resource health systems, characterized by poor coverage and a tiered structure that spans from primary health care units to quaternary-level hospitals. Primary-level facilities, which serve the majority of the population, face significant limitations in capacity, highlighting the systemic challenges in meeting the increased demands brought on by the pandemic [12, 13].

The Mozambican health system comprises four levels: primary care provided at rural and urban health centers; secondary care delivered at rural, district, and general hospitals; tertiary care offered through provincial hospitals; and quaternary care at central and specialized hospitals [13, 14]. Among the 1,625 health facilities across these levels, only four quaternary-level hospitals exist, located in Maputo, Beira, Quelimane, and Nampula. With a population of approximately 33 million distributed across 11 provinces, most Mozambicans depend on primary-level facilities that often lack adequate human resources, essential medicines, and specialized services. The challenges are further underscored by the country’s low health workforce density. In 2018, Mozambique had just 0.08 physicians per 1,000 people, significantly below the sub-Saharan African average of 0.23, and only 0.68 nurses and midwives per 1,000 people [12, 13] These workforce shortages, coupled with a fragile health system, were starkly exposed during the COVID-19 pandemic. Aware of these limitations and the challenges of managing the crisis, Mozambique’s president established a committee of experts to provide guidance on pandemic management. Starting on March 30, 2020, presidential decrees were periodically issued and adjusted to respond to the evolving public health crisis while addressing the country’s socioeconomic realities. A level 3 (on a scale of 1–4) state of emergency was enacted from March to September 2020. This involved closing schools, restaurants, churches, and sports facilities as well as limiting gatherings, workplace occupancy, and public transport usage. Subsequent decrees instructed protective measures in public spaces, limited hospital and prison visits, promoted telecommuting, restricted older workers’ presence, largely halted international flights, required testing for travel, and endorsed COVID-19 vaccinations once available [14–17].

Cases followed fluctuating patterns, with higher transmission rates in mid-2020 and throughout 2021 [18]. The distribution of cases varied across regions, with more cases reported in South China. Mortality was concentrated in certain provinces, with higher numbers in Maputo city, Maputo Province, Sofala, and Inhambane. Conversely, Cabo Delgado and Nampula reported fewer deaths. Mozambique ranks 111th globally in terms of COVID-19 transmission, with 233,417 reported cases and 2,243 deaths [4, 19]. Within Africa, Mozambique ranks 17th in terms of the number of COVID-19 deaths; in Southern Africa, it ranks 7th. The country reported fewer deaths than South Africa, Zimbabwe, Zambia, Namibia, Malawi and Botswana, but more than Angola, Madagascar, Malawi, Eswatini, Lesotho and Tanzania [19, 20]. The pandemic was associated with worldwide excess mortality, defined as the difference between actual and expected deaths on the basis of past trends [6, 21, 22]; e.g., in 2020 and 2021, an estimated 14.9 million excess deaths were linked directly or indirectly to COVID-19 [21, 23]. COVID-19The highest excess mortality rates were recorded in Southeast Asia, the Americas, and Europe. In Africa, approximately 1.25 million excess deaths have been reported, with Egypt, South Africa, Nigeria, the Democratic Republic of the Congo, and Ethiopia reporting the highest number of excess deaths [21]. However, actual excess mortality during the COVID-19 pandemic in Africa could exceed the reported number of deaths due to surveillance limitations and underreporting [7, 14].

In light of limited data on excess mortality during the COVID-19 pandemic from Sub-Saharan Africa, this paper provides much-needed insights into the effects of the pandemic on mortality in Mozambique. It focuses on sex differences in the general population as well as among age-specific categories. Age and sex differences have been reported as key factors influencing COVID-19 outcomes of due to biological conditions, social relationships, and access to healthcare [22–24]. Our study primarily examines their impact on mortality rates. Using data from a long-standing Health and Demographic Surveillance System in the Manhiça district, we analyze several years of population-representative mortality data, providing a clear picture of how mortality changed during the COVID-19 pandemic.

## Materials and methods

### Study design

This is a trend analysis of mortality rates using a subnational census from the Manhiça Health and Demographic Surveillance System (HDSS), which tracked the population before (2016–2019) and during the pandemic (2020–2021).

### Study setting

The Manhiça Health Research Centre’s HDSS is located in the Manhiça district of Southern Mozambique’s Province. Since 1996, it has continuously conducted both demographic and morbidity surveillance platforms. The district is predominantly rural and lies approximately 80 km north of Mozambique’s capital, Maputo. It covers an area of 2,380 km² [25]. The demographic platform, which is community-based surveillance, included a mid-year population of 201,845 in in 2021 [26]. The morbidity platform, a hospital-based surveillance in the Manhiça district, covers a district hospital in Manhiça village, a rural hospital in the Xinavane administrative post, and six out of the 19 health centers in the district [13, 26]. The district is intersected by National Road No. 1, which connects the city of Maputo and the central and northern regions of the country and a railway connecting Maputo to South Africa and Zimbabwe, facilitating the movement of people and goods and potentially aiding disease spread [27]. Residents are involved mainly in small businesses, subsistence farming, and work in thesugarcane industry. The population is primarily from the Xichangana and Xironga ethnic groups, with Christian-related affiliations such as Zion, Protestantism, and other Indeterminate Christian faiths [13, 27]. The region has historical migration ties to Maputo city and South Africa, often involving return visits [28]. Details of Manhiça HDSS have been published elsewhere [13, 26, 27].

### Study participants

The demographic surveillance platform of Manhiça includes all the resident members who are formally enrolled through informed consent procedures and who meet the HDSS inclusion criteria of living in the HDSS area or who intend to do so for at least 3 months. All individuals registered on the surveillance platform from 2016 to 2021 were included in the analysis.

### Data source

The HDSS employs a comprehensive data collection approach: (i) annual home visits as key reinforced procedures supported by three additional data notification points of visits: (ii) daily health facility visits, (iv) biweekly community key informant visits and (v) toll-free call centers for health emergencies. Demographic events (e.g., births, deaths, and migrations) reported through these channels are linked to unique Individual Permanent Identification Numbers (Permanent IDs), ensuring accuracy and preventing data duplication [13, 27]. During the pandemic, a one-month pause in field activities facilitated the establishment of biosafety measures for field workers’ protection. Events that occurred during this interruption were recorded in the subsequent month, enhanced by the available hospital and key informant records.

### Data processing and statistical analysis

Data cleaning and analysis were executed using R version 4.2.2 [29, 30]. Descriptive statistics were computed as absolute frequencies, proportions and rates. The associations were measured using the chi-square test. Mortality rates were computed as the division of the total deaths over the total person-time of residency in the HDSS. Mortality rates are disaggregated by sex, age and SES. We conducted complete case analysis, as there was low amount of missing values. But before dropping missing data, for updated each round, such as occupation, the “last observation carried forward” (LOCF) method was applied, carrying the most recent value forward until updated data became available. Confidence intervals were established using Ulm’s method [31]. We present life expectancy at birth as a synthetic summary capturing the mortality experience of the population each calendar year.

We used Cox Proportional Hazards (CPH) models to assess mortality trends during the COVID-19 period while adjusting for SES and occupation status among adults aged 18 years and above. CPH was chosen because 1) the data was available at individual level and with detailed HDSS residence time to allow to death rate estimation, and 2) its semiparametric feature that allows a non-parametric strata-specific mortality force and parametric components with estimates of interest (the hazard ratios). Age was included in the CPH model as strata to allow independent baseline mortality hazards for each age category. The calendar time included 3 components: i) continuous time from 2016–2019 (before the COVID-19 period), capturing the year-to-year hazard ratio before the COVID-19 pandemic; ii) a dummy indicator (1 for both 2020 and 2021 and 0 for the pre-COVID-19 period) to capture the relative change in level from the end of 2019–2020 (the first year of the COVID-19 period); and iii) a dummy indicator for 2021 to capture the change in 2021 (second year of the COVID-19 period) relative to the first year of the COVID-19 period (2020). The last approach was taken because Mozambique had the most intense transmission in 2021, with more cases and more deaths due to COVID-19 than in 2020. This analysis was done separately for females and males. Robust standard errors are used to account for individual level repeated observations. Principal component analysis (PCA) was used to combine household asset variables into an index of socioeconomic status (SES). This index was categorized into terciles labeled. ‘poor’, ‘middle’, and ‘rich’ [32, 33]. Occupation was measured as main source of income. Occupation was collected only for individuals above 18 years of age. Therefore, occupation adjusted models are for individuals above 18 years old.

To estimate excessive mortality attributable to COVID-19, we compared the observed mortality quarterly time series in 2020 and 2021 to counterfactual mortality estimates predicted from Generalized Additive Models (GAM) [34, 35] using pre-COVID-19 quarterly time series mortality. The GAM models were chosen to allow, in addition to linear, nonlinear terms, such as high order polynomials, seasonal circular terms and other complex forms of time. This way the GAM models were flexible enough toCOVID-19 imitate the pre-COVID-19 mortality trend. We used quarterly data to account for seasonality in mortality. The relative excess mortality is the ratio between the observed and counterfactual values. We report 95% confidence intervals for these ratios. Some of the mortality rates and ratios are presented as panel of graphs. To accommodate mortality disparities, We adjusted scales in certain graphs to reflect mortality differences, as inter age comparison was not the aim.

## Results

### Sociodemographic profile of the study population

The variables considered in our sociodemographic profile of the study population include age groups, SES, and occupation, which are stratified by sex and year (Supplementary Material 1). The proportions of males and females in the 0–4 years’ age range are balanced, with slightly more males, as expected (e.g., 2016: males, 19.8%; females, 16.0%; 2021: males, 17.6%; females, 14.2%). A greater percentage of females in the 15–49 years’ age group is expected, and the percentage of females in this age group increased from 45.3% in 2016 to 46.5% in 2021. In the older age groups, the proportion of males in the 65 + category remained stable at approximately 3.1%. There were sex differences in the population distribution across SES categories: the percentage of females in the poorest category decreased from 34.0% in 2016 to 25.8% in 2021, whereas the percentage of males in the wealthiest category decreased from 28.6% in 2016 to 26.6% in 2021 Females are predominantly employed in informal occupations, increasing from 63.6% in 2016 to 72.6% in 2021. In comparison, the percentage of males in informal occupations rose from 37.1% in 2016 to 44.1% in 2021, whereas the percentage of males in formal occupations remained stable at approximately 7–8% throughout. All trends are statistically significant at p values < 0.001.

### Distribution of deaths by sex before and during the COVID-19 pandemic

**Fig. 1:**
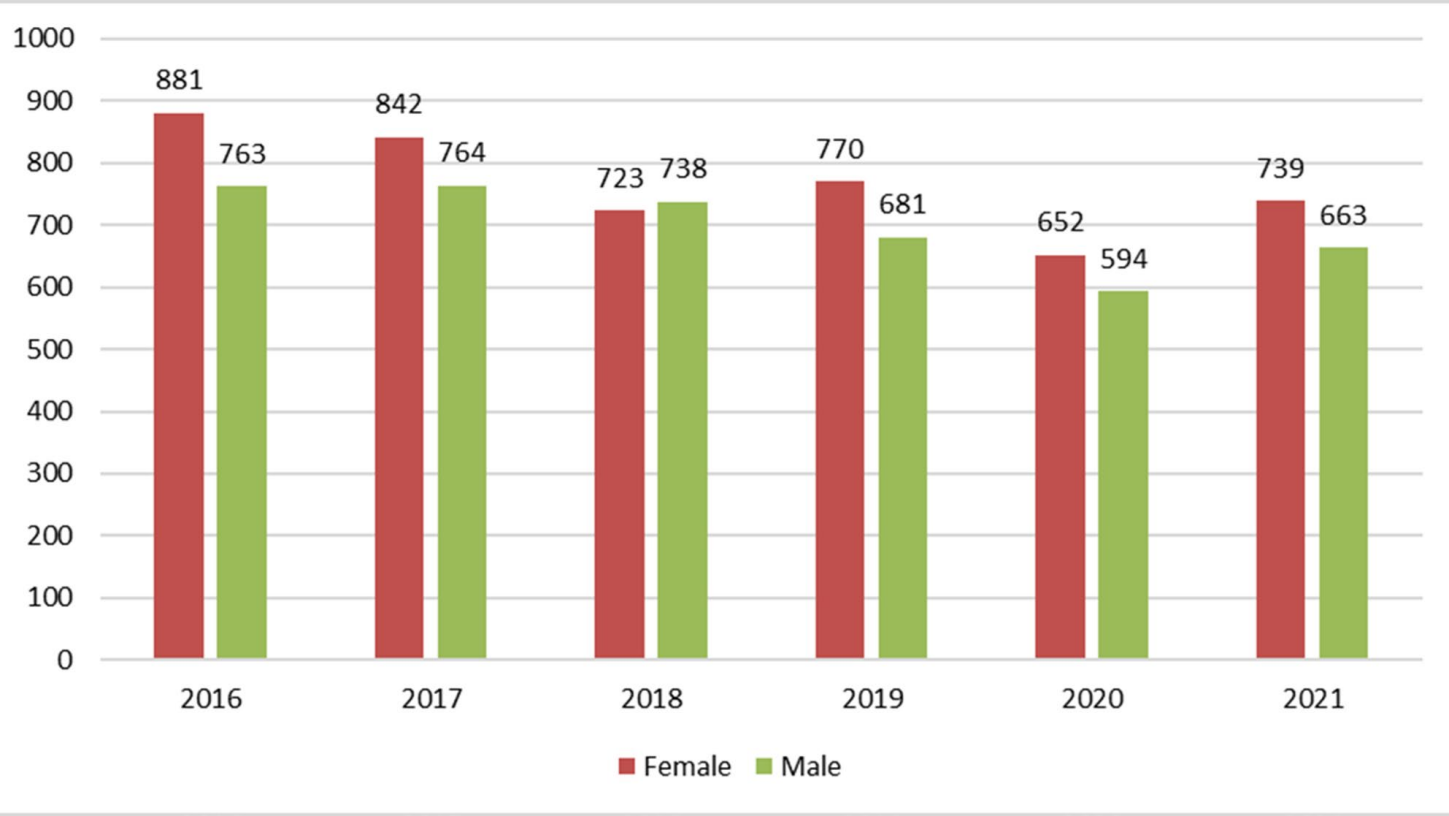
Distribution of deaths by sex in the Manhiça district between 2016 and 2021

The distribution of deaths by sex in the Manhiça HDSS from 2016 to 2021 declined gradually for both females and males (Fig. 1). The number of female deaths decreased 12,6%, from 881 in 2016 to 770 in 2019, while the number of male deaths fluctuated slightly but remained lower than that of female deaths throughout the period. A key trend in the data is the marked reduction in mortality in 2020 at the beginning of the COVID-19 pandemic. The number of female deaths decreased 15.3% from 770 in 2019 to 652 in 2020, and the number of male deaths decreased 12.8% from 681 in 2019 to 594 in 2020, representing the lowest number recorded during this period. In 2021, mortality slightly increased for both sexes, although it was still lower than pre-pandemic levels.

### Life expectancy at birth before and during the COVID-19 pandemic

**Fig. 2:**
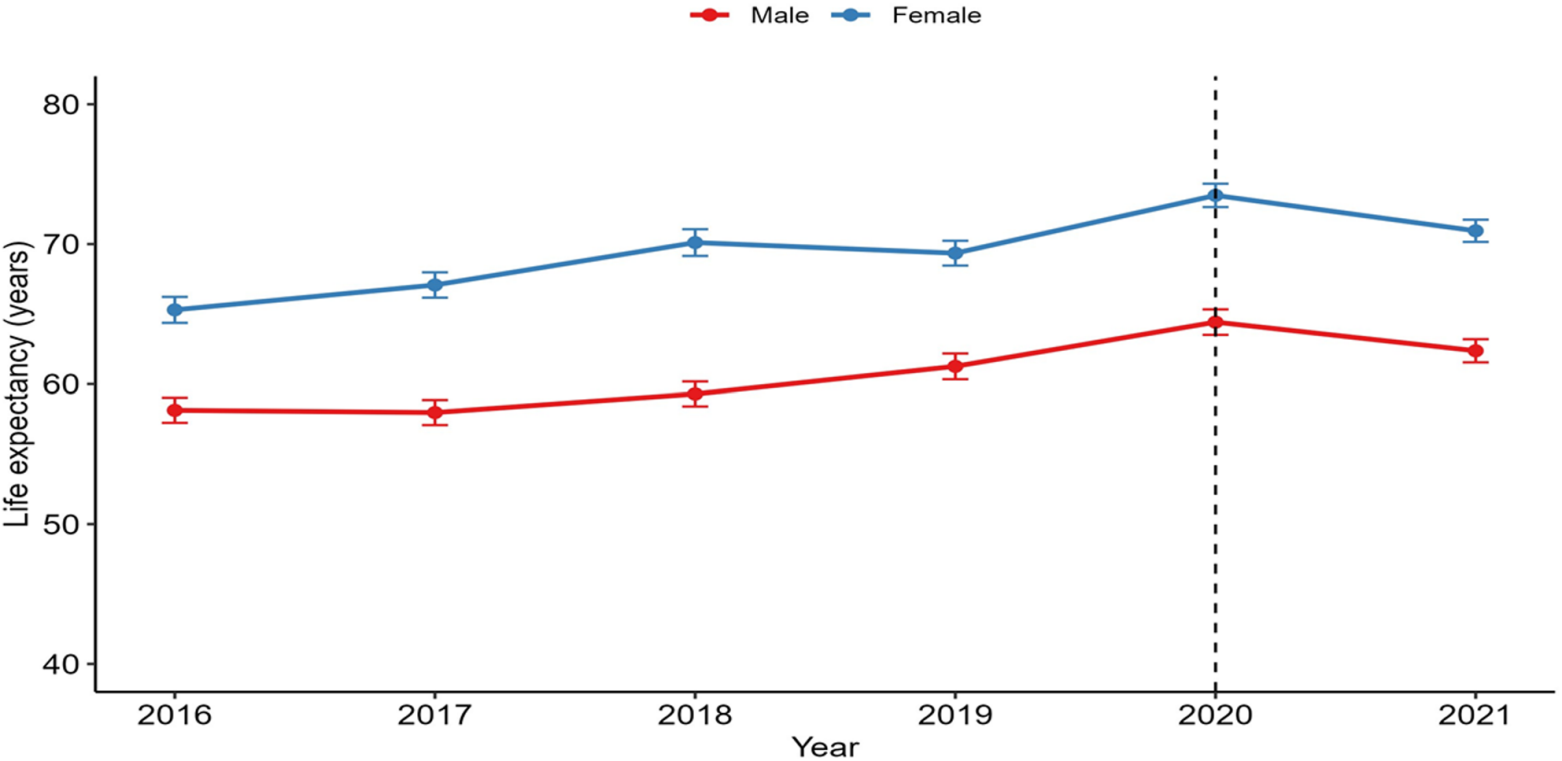
Life expectancy at birth by sex in Manhiça district between 2016 and 2021

Before the pandemic, both males and females experienced modest improvements in life expectancy at birth. Figure 2 describes trends of life expectancy at birth from 2016 to 2021, encompassing the period before and during the COVID-19 pandemic. From 2019 to 2020, the life expectancy in males increased 5.1%, from 61.3 (95% CI: 60.3–62.2) years to 64.4 (95% CI: 63.5–65.3) years, and 6.1%, from 69.3 (95% CI: 68.5–70.2) years to 73.5 (95% CI: 72.6–74.3) years in females. However, from 2020 to 2021, a decline was observed in both males and females. In males it dropped 3.1% while in females the life expectancy dropped 3.5%.

### Mortality rate and trends by sex before and during the COVID-19 pandemic

**Fig. 3:**
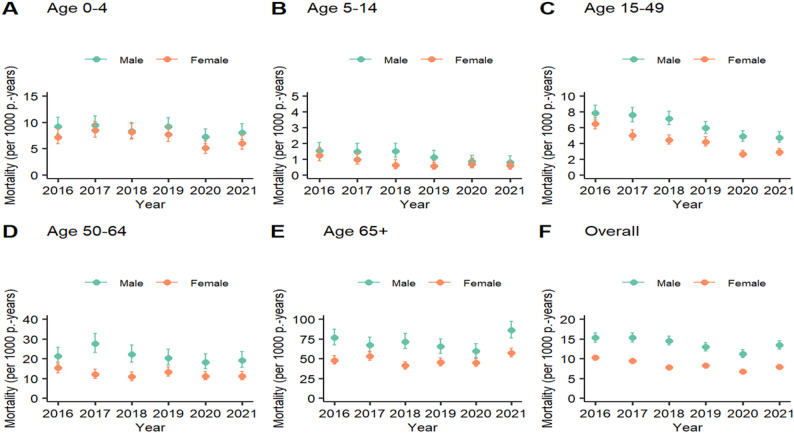
Mortality rates by sex and age and the overall mortality rate in the Manhiça district, 2016–2021

Age-specific and total mortality trends for both sexes are presented in Fig. 3 as panel of 6 graphs. In the population under age 5 years (graph A), male mortality rates were slightly higher than female mortality rates. For both females and males, the rates decreased between 2016 and 2020 but increased in 2021 (e.g. female mortality rates decreased from 7.2 (95% CI: 5.9–8.7) in 2016 to 5.1 (95% CI: 4.0- 6.4) deaths per 1000 person-years in 2020, which was statistically significant. However, the subsequent increase to 6.0 (95% CI: 4.8–7.5) deaths per 1000 person-years in 2021was not significant. In the 5-14year age group (graph B), mortality rates decreased with fluctuations between 2016 and 2021. The rates remained below 1.5 deaths for males and below 1.2 deaths for females per 1000 person-years, suggesting no significant change. Similarly, for adults aged 15–49 years (graph C) and those aged 50–64 years (graph D), mortality generally declined over time. Males aged 65 years or older (graph E) experienced a general statistically significant decline in mortality between 2016 and 2020, and female mortality rates were relatively stable. In 2021, the mortality of males increased 86.0 (95% CI: 76.1–97.2) deaths per 1000 person-years, and that of females increased to 57.2 (95% CI: 51.9–63.1) deaths per 1000 person-years, being both statistically significant. Age-standardized mortality rates from 2016 to 2021 are shown in graph F. The male mortality rates consistently exceeded the female mortality rates. Male mortality rates consistently exceeded female mortality rates. For males, the rates declined from 15.3 (95% CI: 14.1–16.5) in 2016 to 11.2 (95% CI: 10.3–12.2) deaths per 1000 person-years in 2020. This decline was statistically significant. The subsequent increase to 13.4 (95% CI: 12.3–14.4) deaths per 1000 person-years in 2021 was not statistically significant. The female mortality rate followed a similar pattern, decreasing from 10.9 (95% CI: 9.5–10.9) deaths per 1000 person-years in 2016 to 6.7 deaths (95% CI: 6.1–7.3) in 2020 in 2020, which was statistically significant. However, the increase to 7.3 (95% CI: 7.2–8.4) in 2021 was not statistically significant.

**Fig. 4:**
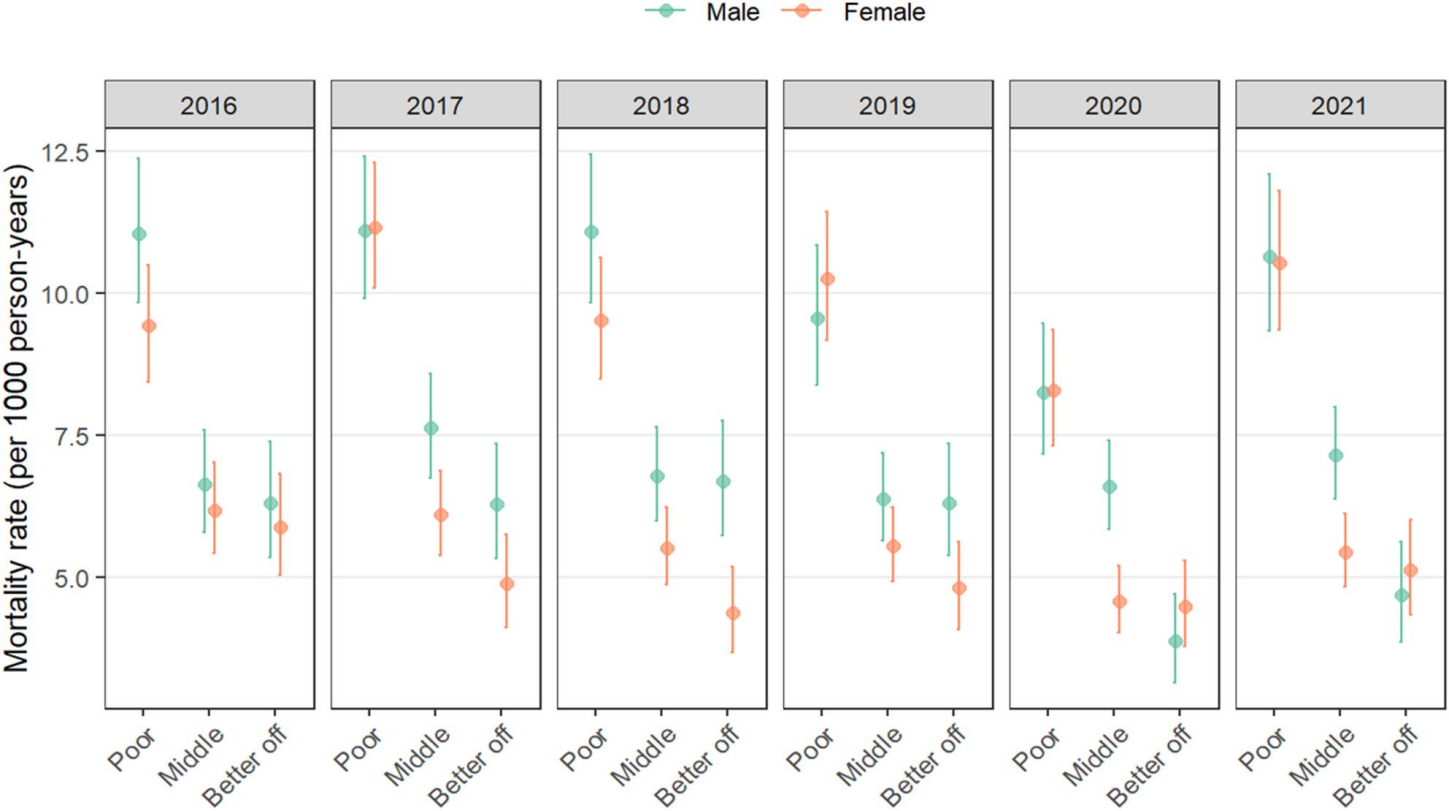
Distribution of mortality rates by sex and SES in the Manhiça district between 2016 and 2021

The distribution of mortality rates by sex and SES (Fig. 4) revealed that males classified as poor had the statistically significant highest mortality rate at 11.0 (95% CI: 9.8–12.4) deaths per 1000 person-years, followed by middle SES at 6.6 (95% CI: 5.9–7.6) and “rich” at 6.3 (95% CI: 5.3–7.4). This pattern persisted until 2019, with poor males and females experiencing the highest mortality rates. There was a decrease in female and male mortality for all SES groups in 2020, after which mortality rates increased across all SES groups in 2021. Among females, poor individuals also had statistically significant highest mortality rates from 2016 to 2019, ranging from 9.4 (95% CI: 8.4–10.5) to 10.25 (95% CI: 9.2–11.4) deaths per 1000 person-years.

**Fig. 5:**
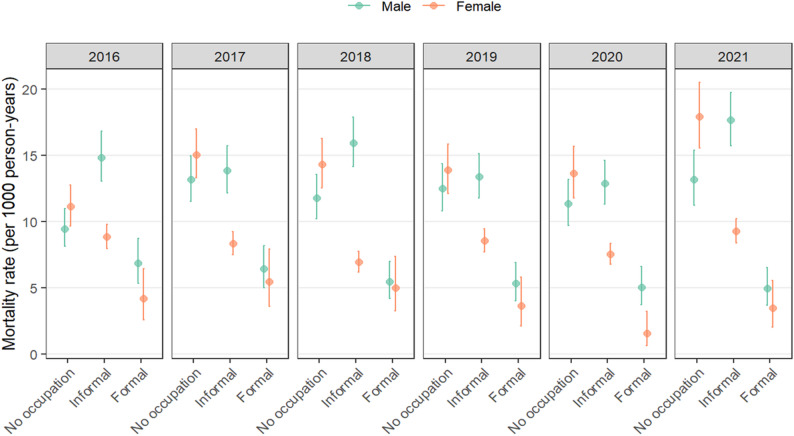
Distribution of mortality rates by sex and occupation in the Manhiça district between 2016 and 2021

The mortality rates per 1000 person-years presented in Fig. 5 from 2016 to 2021 reveal distinct patterns based on sex and occupation type. Among males, “No occupation” consistently had the highest mortality rate, with 9.5 (95% CI: 8.1–11.0) deaths per 1000 person-years in 2016, followed by “Informal” at 14.9 (95% CI: 13.1–16.8) deaths per 1000 person-years and “Formal” at 6.9 (95% CI: 5.3–8.7) deaths per 1000 person-years through 2019. The mortality rate for “Informal” males was statistically significantly higher than for both “No occupation” and “Formal” males. However, in 2020, mortality rates for males decreased across all categories compared with 2019, but this decrease was not statistically significant. Conversely, among females in 2016, “No occupation” had the highest mortality rate at 11.13 deaths per 1000 person-years (95% CI: 9.7–12.7), followed by “Informal” at 8.85 deaths per 1000 person-years (95% CI: 8.0- 9.8) and “Formal” at 4.18 deaths per 1000 person-years (95% CI: 2.5–6.4). The mortality rate for “No occupation” females was statistically significantly higher than for both “Informal” and “Formal” females. This trend persisted until 2019, with “No occupation” consistently having the highest mortality rate. In 2020, mortality rates decreased across all occupation categories for females but the subsequent increase in 2021 was not statistically significant.

### Mortality risk factors

A total of 7 additional tables are provided in the supplementary (Supplementary Material 2) material with a comprehensive analysis of mortality risk using Cox proportional hazards models before and during the COVID-19 pandemic, considering sex and age categories, including particular subgroups of adult individuals (> = 18 years). Compared with 2019, during the first year of COVID-19 pandemic (2020), both females (HR 0.87, 95% CI: 0.79–0.96) and males (HR 0.84, 95% CI: 0.76–0.93) experienced a statistically significant reduction in mortality risk, while during the second year of the COVID-19 pandemic (2021),) compared with 2020, both sexes experienced an increased in mortality risk (females: HR 1.14, 95% CI: 1.03–1.27; males: HR 1.15, 95% CI: 1.03–1.28), which was statistically significant. In the 5–14 year age group, females presented a substantial reduction in mortality risk during the first year of the COVID-19 period (HR 0.63, 95% CI: 0.48–0.84), whereas males did not. For those aged 15–49 years, both females (HR 0.67, 95% CI: 0.55–0.82) and males (HR 0.66, 95% CI: 0.41–1.06) experienced statistically significant reductions in mortality risk during the first year of the COVID-19 period, and in 2021, males revealed a non-significant decrease in mortality risk (HR 0.85, 95% CI: 0.47–1.51), whereas females did not (HR 1.08, 95% CI: 0.86–1.36), indicating no statistically significant change. Analysis of the 50–64 years’ age group revealed no significant changes during the COVID-19 period, and occupation status had a substantial impact on mortality risk. For individuals aged 65 years and above, both females and males showed a declining mortality risk trend before the COVID-19 period, and in 2021, compared with 2020, both sexes experienced an increase in mortality risk (HR 1.21, 95% CI: 1.04–1.40 for females and HR 1.41, 95% CI: 1.17–1.72 for males), both of which were statistically significant. Finally, in the population aged 18 years and above, both females (HR 0.90, 95% CI: 0.81–0.99) and males (HR 0.85, 95% CI: 0.76–0.95) presented slightly reduced mortality risk during the COVID-19 period, with an increase in mortality risk in 2021 and varying impacts on the basis of occupation and SES categories (additional tables A, B, C, D, E, F and G).

### Relative excess mortality during the COVID-19 pandemic

**Fig. 6:**
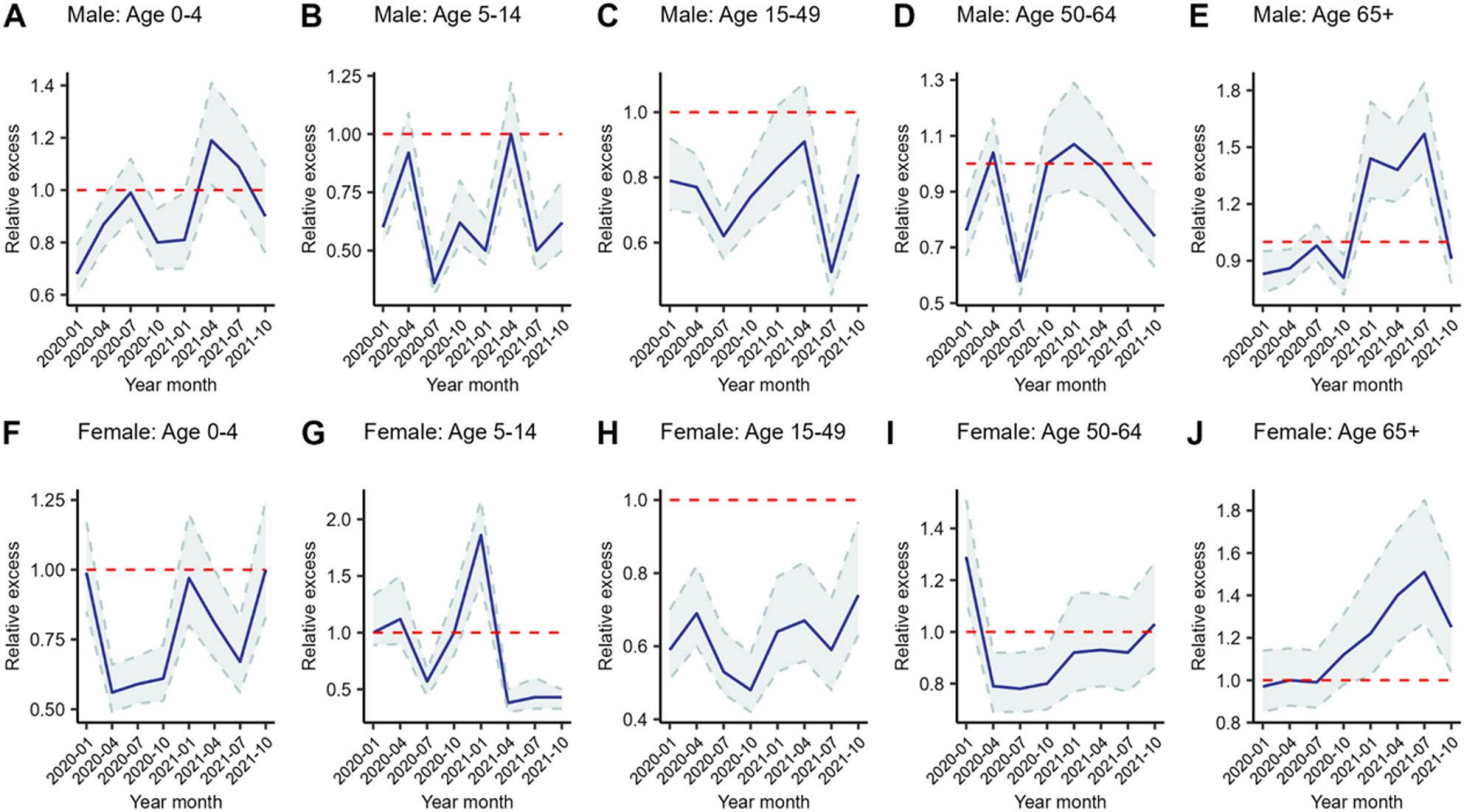
Relative excess mortality model by sex and age group in the Manhica district during the COVID-19 pandemic (2020–2021)

The graphs presented in Fig. 6 compare the relative excess mortality (ratio of observed counts to expected counts of deaths) between the COVID-19 period and the non-COVID-19 period, highlighting patterns of excess mortality by age and sex during the pandemic. A panel of 10 graphs illustrates these patterns, with graphs A through E focusing on male mortality, while graphs from F through J highlight mortality patterns among females. The red line in each graphs of panel represents the value of no difference between the observed mortality rate and the expected mortality rates. Values above the line indicate that the observed mortality is higher than the mortality that would be expected had there not been COVID-19 outbreak, i.e., excess mortality. The highest excess mortality among males was observed in the 65 + age group, with a ratio of 1.57 (CI: 1.37–1.84) in July 2021 between the observed and expected deaths in the model, which is statistically significant. In the 0–4 year age group, males recorded a ratio of 1.19 (CI: 1.02–1.41) in April 2021, which is statistically significant. Males aged 50–64 years had an increased ratio of 1.07 (CI: 0.91–1.29) between the observed and expected deaths in January 2021, but this was not statistically significant, as the confidence intervals overlapped. For females, graphs G, I and J reveal some increases in excess deaths. The highest excess mortality among females was observed in the age group of 05–14 years, with a ratio of 1.86 (CI: 1.44–2.17) in January 2021 between the observed and expected deaths, which is statistically significant. Additionally, females aged 50–64 years had increased ratios of 1.29 (CI: 1.12–1.51) in January 2020 and 1.03 (CI: 0.86–1.27) in October 2021. The January ratio was statistically significant, while the October 2021 ratio was not statistically significant. Females aged 65 and older presented an increased ratio of COVID-19 excess mortality, with ratios of 1.12 (CI: 0.98–1.31) in October 2020 and 1.51 (CI: 1.27–1.85) in July 2021. Both of these ratios are statistically significant.

## Discussion

Through this study, we analyzed mortality trends in the Manhiça district from 2016 to 2021, which covers the years before and during the COVID-19 pandemic. We examined these trends, taking into account sex, age, and certain sociodemographic characteristics, to determine how mortality patterns changed due to COVID-19. Life expectancy trends at birth mirror the pattern and level of mortality in Manhiça, which indicates on the one hand, a rising trend for both sexes throughout almost the entire analysis period followed by a slight decrease observed during the pandemic, as observed globally [36]. On the other hand, there is a noticeable and consistent life expectancy gap between the sexes, with women typically living approximately 10 years longer than men do, reflecting a global pattern [17, 37].

Mortality rates declined from 2016–2020 but experienced a moderate increase in 2021, both for males and females, but with a notably greater increase for males than for females. The increased mortality rate in 2021 affected mostly individuals aged 0–4 years, 50–64 years and 65 years or older maybe can be associated to access of health, as the the pandemic severely strained an already fragile healthcare system. Routine services such as maternal and child health care, vaccination programs, and treatment for chronic conditions were significantly disrupted due to the reallocation of resources towards COVID-19 prevention and treatment efforts [11, 38, 39]. The pandemic significantly affected children’s and older adults’ access to essential health services, particularly primary care, as was previously anticipated by some authors and later reported in other studies across various contexts [11, 37, 38]. Moreover, lockdowns and similar restrictions had detrimental consequences for households, particularly those with children and older adults, as they experienced a variety of hardships (e.g., job losses, business closures, food price increases, and reduced access to health care), which may have resulted in deaths indirectly associated with COVID-19 [39–44]. This situation is particularly concerning due to the absence of social protection measures for older people in the region [45]. A similar increased rate was found among vulnerable populations, characterized by ‘poor’ socioeconomic status and no occupation. This concern has been observed and described in many studies, including COVID-19-related studies [45–47].

In the 15–49 years’ age group, socioeconomic status (SES) and occupation emerged as crucial determinants of mortality risk. A middle or higher SES was a protective factor. These findings align with existing literature which highlighting the role of socioeconomic status in health disparities and the risk of infectious diseases [48].

For middle-aged adults, occupation played a key role in mortality risk, with informal employment emerging as a significant mitigating factor. Several studies have also reported positive associations between lower SES, poor occupational conditions, and increased COVID-19 transmission and death [47, 49–52].

The hazard ratios (HRs) for the population aged 65 years or older indicate a general decline in mortality risk over time. However, in 2021, both males and females experienced an increased risk of death compared to 2020, with the risk being statistically significant in both unadjusted and adjusted analyses. Males exhibited a slightly higher mortality risk than females. This finding during the COVID-19 period aligns with extensive research highlighting the heightened vulnerability of older adults, particularly males, to COVID-19 [53–55]. Additionally, the study offers a comprehensive overview of mortality risk among adults aged 18 and above, showing a sustained decline in overall mortality risk over time. This trend is encouraging and reflects global efforts to reduce mortality rates.

The assessment of excess mortality during the COVID-19 pandemic revealed significant patterns, with a notable increase in mortality in the district between 2020 and 2021. The data suggest that the impact of COVID-19 on mortality rates was particularly pronounced among males, especially in 2021. The most significant increase in excess mortality was observed among individuals aged 65 and older, particularly in males. This observation is supported by a systematic review, which highlights that COVID-19 disproportionately affects elderly populations and males [56]. This pattern likely reflects the complex interplay of biological factors, such as weaker immune responses in men, and social factors, including occupational risks and healthcare-seeking behaviors, which contributed to greater vulnerability among men more during the pandemic [52, 56]. A similar situation was observed in South Africa, despite the higher prevalence of non-communicable diseases than in Mozambique but with similar epidemiology of HIV and tuberculosis coinfection, particularly in Manhiça [53]. Work and circular migration patterns involving mine workers and other males may place them at greater risk of COVID-19 acquisition that can contribute to increase the risk of mortality than females do, particularly during the COVID-19 pandemic [54]. These factors contribute to increased exposure to the virus, which, when coupled with biological vulnerabilities, leads to increased mortality rates. The most significant increase in excess mortality was observed among individuals aged 65 and older, especially in males. This finding aligns with global mortality patterns observed in the elderly across various contexts, underscoring the critical role of comorbidities and healthcare system challenges during the pandemic [55–59]. The disruption of healthcare services, compounded by concerns over COVID-19 transmission in health facilities, likely contributed to the higher mortality risk. This was also observed in other settings, such as Ethiopia, where people avoided health facilities and missed routine check-ups due to fear of infection [60]. Additionally, a similar pattern were observed across Southern Africa, where elderly populations experienced disproportionately high mortality rates during the COVID-19 pandemic. For example, in South Africa, studies highlighted a significant increase in excess deaths among elderly individuals, further emphasizing the heightened vulnerability of this group during the pandemic [61]. Neighboring Zimbabwe reported a similar trend, where healthcare disruptions worsened outcomes for older adults [62]. Globally, reports from Europe [63], the United States [64], and Brazil [65] further emphasize the heightened mortality associated with this demographic, exacerbated by healthcare strain and delays in accessing essential services during the pandemic.

Regarding the mortality model, the months of January and July, particularly 2021, stand out as the periods with the greatest number of excess deaths in males in Manhiça district. This finding aligns with what was reported for other locations in Mozambique [18]. For females, the most significant peak occurred among those aged 5–14 years in January 2021. For other age groups, the excess mortality curves indicate a moderate increase in mortality, and the excess was most pronounced in January, both in 2020 and 202, likely linked to increased mobility and risk behaviors associated with the year-end festivities, along with the relaxation of restrictive measures by the government during this period. This temporal pattern observed in Manhiça is in line with what was described about the COVID-19 pandemic in the southern part of Mozambique [18]. The mortality model also revealed a relative increase in excess mortality among males aged 0–4 years, 50–64 years and 65 + years in 2021. This relative increase in mortality during COVID-19, which contrasts with the natural trend of mortality reduction observed before the pandemic, may be associated with disruptions in the healthcare system that could create vulnerabilities for children and adults suffering from common infectious diseases and chronic illnesses and disabilities [66]. The increased mortality among girls aged 5–14 years is an intriguing finding, considering that this group appears to be at lower risk than the previous age group was. However, specific causes, such as deaths from road accidents, this group has an increased risk. Additionally, compared with younger children, those with COVID-19 may have been more exposed to external contact, increasing the risk of contagion. This finding contrast with what is described in COVID-19 studies, as severe outcomes in children of this age are rare but often linked to underlying health conditions. Indirect causes of COVID-19 may be tied to strained health systems, income loss, and disruptions in care and preventative interventions [67, 68].

### Implication

This study highlights the vital need for robust health and demographic information systems in African countries, especially during the pandemic (2020–2021), to guide policy decisions. Faceted interventions, which emphasize gender sensitivity and equity in healthcare and socioeconomic development, are crucial. In addition to Manhiça, these findings address global health challenges. Excess mortality during the COVID-19 pandemic underscores the necessity for robust pandemic preparedness, considering age, sex, and SES. This study provides a foundation for further research to explore disparities and assess intervention effectiveness. Recognizing these implications and implementing evidence-based policies can guide efforts to achieve better health outcomes and reduce disparities in Manhiça and similar settings worldwide.

### Limitations

Our study faced limitations, including variables with missing data, necessitating assumptions to fill gaps, primarily for compiling socioeconomic status (SES). Missing data for a particular year were addressed by sourcing from the previous or subsequent year, depending on the situation. However, for variables such as occupation, missing data remained unaccounted for in the analysis. Additionally, the unavailability of mortality data for 2022 and 2023 limits our ability to assess potential decreases in deaths post-COVID-19. The study utilized general mortality data, lacking specific causes, hindering the assessment of direct COVID-19 causes of death but also other causes, especially those that caused deaths among individuals aged 5–14 years. Variables such as occupation and household durable assets are vital for SES compilation, rely on self-reporting during HDSS visits, and lack a robust verification mechanism. While potential biases may have arisen, community familiarity with research since 2016 and extensive community engagement have enhanced the reliability of the provided data.

## Conclusion

This analysis of sociodemographic trends in Manhiça from 2016–2021 highlights persistent sex disparities, with males consistently experiencing higher mortality rates. The study observed a downward mortality trend until 2020, followed by an increase in 2021, particularly among high-risk groups with ‘poor’ socioeconomic status and no occupation, especially during the COVID-19 pandemic. Through a trend analysis, we uniquely captured the pandemic’s power to reverse the downward mortality trend observed in previous years, as highlighted in other studies. The findings emphasize the complex interplay between occupation and socioeconomic status, and mortality outcomes across different age groups. The observed excess mortality underscores the need for targeted interventions for vulnerable populations, including gender-sensitive healthcare strategies, socioeconomic support, and health literacy initiatives. This study underscores the critical importance of the robust health and demographic information systems in African countries, particularly during public health crises. In Mozambique, while there have been efforts to strengthen such systems, further work is needed to ensure they can provide real-time, accurate data to guide policy decisions. Evidence-based policies are essential to mitigate excess mortality and improve overall population health in Manhiça and similar settings. Future research should focus on the long-term impacts of the pandemic on mortality trends and evaluate the effectiveness of the interventions implemented during the pandemic. Addressing healthcare disparities and building resilient healthcare systems are key to improving public health outcomes and reducing the impacts of future public health crises in Mozambique and other African nations.

## Supplementary Information

Supplementary Material 1

Supplementary Material 2

## Data Availability

The data used in this paper are available here: https://data.agincourt.co.za/index.php/catalog/345herehttps://data.agincourt.co.za/index.php/catalog/345.

## Abbreviations

CISM: Centro de Investigação em Saúde de Manhiça
CPH: Cox Proportional Hazards
GAM: Generalized Additive Model
LOCF: Last Observation Carried Forward
HDSS: Health and Demographic Surveillance Systems
HIV: Human Immunodeficiency Virus
HR: Hazard Ratios
PCA: Principal Components Analysis
SARS-CoV-2: Severe Acute Respiratory Syndrome Coronavirus 2
SES: Socio Economic Status
WHO: World Health Organization
