# Supplementary material for: Mortality before and during the COVID-19 pandemic in Manhiça district, Southern Mozambique
Source: Popul Health Metr. 2026 Mar 18;23(Suppl 2):76. doi: 10.1186/s12963-025-00449-y (PMC13001221; doi:10.1186/s12963-025-00449-y)
Supplement: Supplementary file 1 — Supplementary Material 1 [file 12963_2025_449_MOESM1_ESM.docx]

**Table 1:** Sociodemographic characteristics of the study population in Manhiça between 2016 and 2021

| **Variables** | **Categories** | **2016** | | **2017** | | **2018** | | **2019** | | **2020** | | **2021** | | **P value** |
| --- | --- | --- | --- | --- | --- | --- | --- | --- | --- | --- | --- | --- | --- | --- |
|  |  | **Male N=91,080** | **Female N=111,297** | **Male**  **N=96,060** | **Female N=116,739** | **Male N=99,404** | **Female N=120,914** | **Male N=101,365** | **Female N=123,611** | **Male N=100,931** | **Female N=123,385)** | **Male N=99,739)** | **Female (121,724)** |  |
| Age groups | 0-4 | 19.8% | 16.0% | 19.4% | 15.7% | 19.1% | 15.5% | 18.8% | 15.1% | 18.2% | 14.8% | 17.6% | 14.2% | <0.001 |
|  | 5-14 | 30.8% | 25.4% | 30.7% | 25.6% | 30.8% | 25.7% | 31.0% | 25.7% | 31.4% | 25.8% | 31.5% | 25.7% |  |
|  | 15-49 | 41.1% | 45.3% | 41.7% | 45.6% | 42.1% | 45.9% | 42.2% | 46.2% | 42.3% | 46.3% | 42.7% | 46.5% |  |
|  | 50-64 | 5.2% | 7.3% | 5.1% | 7.3% | 5.0% | 7.3% | 5.0% | 7.3% | 5.0% | 7.4% | 5.1% | 7.6% |  |
|  | 65+ | 3.2% | 6.0% | 3.1% | 5.8% | 3.0% | 5.6% | 3.0% | 5.6% | 3.0% | 5.7% | 3.1% | 5.9% |  |
|  |  |  |  |  |  |  |  |  |  |  |  |  |  |  |
| SES | Poor | 32.6% | 34.0% | 32.4% | 33.4% | 29.1% | 29.8% | 27.2% | 28.1% | 27.2% | 28.0% | 25.5% | 25.8% | <0.001 |
|  | Middle | 38.8% | 37.8% | 40.3% | 39.6% | 43.0% | 42.6% | 45.4% | 44.8% | 45.7% | 45.4% | 47.6% | 47.6% |  |
|  | Rich | 28.6% | 28.2% | 27.4% | 27.0% | 27.9% | 27.6% | 27.4% | 27.1% | 27.1% | 26.6% | 26.9% | 26.6% |  |
|  |  |  |  |  |  |  |  |  |  |  |  |  |  |  |
| Occupation | No occupation | 41.2% | 29.2% | 38.9% | 26.8% | 36.3% | 25.2% | 34.7% | 24.2% | 34.0% | 22.2% | 31.1% | 19.6% | <0.001 |
|  | Informal | 37.1% | 63.6% | 38.2% | 65.9% | 39.5% | 67.6% | 41.8% | 68.9% | 42.8% | 71.0% | 44.1% | 72.6% |  |
|  | Formal | 21.7% | 7.2% | 22.9% | 7.3% | 24.2% | 7.3% | 23.5% | 6.9% | 23.2% | 6.8% | 24.8% | 7.9% |  |

**Note:** Data source: Manhiça Health and Demographic Surveillance System data. Missing values were removed from the table. Associations were examined via χ^2^ tests.
